# Preoperative profiles of plasma amino acids and derivatives distinguish periampullary cancer and benign disease

**DOI:** 10.1186/s12885-024-12320-8

**Published:** 2024-05-03

**Authors:** Stina Margrethe Stålberg, Laxmi Silwal-Pandit, Nasser Ezzatkhah Bastani, Daniel Johan Hammer Nebdal, Ole Christian Lingjærde, Bjørn Steen Skålhegg, Elin Hegland Kure

**Affiliations:** 1 Department of Cancer Genetics, Institute for Cancer Research, Oslo University Hospital, Oslo, Norway; 2 Department of Natural Sciences and Environmental Health, University of South-Eastern Norway, Bø i Telemark, Norway; 3 Department of Pathology, Skien Hospital, Vestfold og Telemark, Norway; 4 Division for Molecular Nutrition, Institute for Basic Medical Sciences, University of Oslo, Oslo, Norway; 5 Department of Computer Science, University of Oslo, Oslo, Norway

**Keywords:** Periampullary cancer, Metabolite, Amino acid, Blood plasma, PDAC

## Abstract

Periampullary cancers, including pancreatic ductal adenocarcinoma, ampullary-, cholangio-, and duodenal carcinoma, are frequently diagnosed in an advanced stage and are associated with poor overall survival. They are difficult to differentiate from each other and challenging to distinguish from benign periampullary disease preoperatively. To improve the preoperative diagnostics of periampullary neoplasms, clinical or biological markers are warranted.

In this study, 28 blood plasma amino acids and derivatives from preoperative patients with benign (*N* = 45) and malignant (*N* = 72) periampullary disease were analyzed by LC-MS/MS.

Principal component analysis and consensus clustering both separated the patients with cancer and the patients with benign disease. Glutamic acid had significantly higher plasma expression and 15 other metabolites significantly lower plasma expression in patients with malignant disease compared with patients having benign disease. Phenylalanine was the only metabolite associated with improved overall survival (HR = 0.50, CI 0.30–0.83, *P* < 0.01).

Taken together, plasma metabolite profiles from patients with malignant and benign periampullary disease were significantly different and have the potential to distinguish malignant from benign disease preoperatively.

**Supplementary Information:**

The online version contains supplementary material available at 10.1186/s12885-024-12320-8.

## Background

Periampullary cancer is a collection of cancers located in the surroundings of ampulla of Vater, in the head of the pancreas, the duodenum and the distal bile duct. The cancers are all serious malignancies with different frequency, prognosis, and treatment with pancreatic ductal adenocarcinoma (PDAC) being the most frequent (> 80%) and with the poorest outcome. The ampullary cancer which form in the ampulla accounts for 6–9% of the periampullary malignancies. Ampullary cancer usually obstructs the bile duct and may therefore present symptoms and be diagnosed at an earlier stage compared to other periampullary cancers. Hence, ampullary cancers tend to have a better prognosis [1–4].

Surgical resection offers a potential cure for patients with periampullary cancers. However, due to locally advanced and/or metastatic disease, the majority of these patients are not eligible for upfront curative surgery. Some patients with locally advanced cancer may be offered surgery after receiving neoadjuvant therapy with the attempt to shrink and make the tumor more accessible [5]. The guidelines for neoadjuvant therapy are different for the four periampullary cancers; patients with pancreatic cancer is the only periampullary carcinoma recommended to receive neoadjuvant treatment [6–9]. To distinguish between the different periampullary cancers preoperatively is challenging. In a study from 2021, up to 30% of periampullary cancers were found to have been misdiagnosed, of which 16% were clinically relevant affecting the opportunity to receive neoadjuvant therapy [9]. Moreover, the clinical work-up that include clinical evaluation, radiology, and biochemistry to differentiate periampullary cancers and benign disease is inadequate. Consequently, 5–10% of the patients undergo surgery for suspected malignancy only to receive a benign diagnosis after the pathology evaluation [10–13]. To be able to identify the periampullary neoplasms that will benefit from neoadjuvant chemotherapy and surgery, preoperative clinical or biological markers are warranted.

Over the last decade, several studies have shown promising results by using blood-based metabolites as a diagnostic tool to differentiate periampullary cancers from benign disease [14–19]. These studies have presented models with a few to several metabolites to separate cancer from benign disease with better sensitivity and specificity than the established biomarker Cancer Antigen 19-9 (CA19-9). Most studies have compared the metabolite profile of one of the four periampullary cancers to the profiles of healthy controls or patients with clinically benign disease. This approach disregards the heterogeneity of periampullary cancers and does not address the clinical problem of distinguishing these diseases preoperatively. In addition, the benign controls, either healthy volunteers or patients with clinically benign disease are often clinically distinct from cancer. Their metabolite profiles may not be representative of the profiles from benign patients that undergo surgery for a suspected malignant disease.

Amino acids, which are metabolites crucial for the survival and growth of all cell types, undergo substantial changes during reprogrammed metabolism in cancer. Amino acids and close derivatives are building blocks for proteins, can be fueled as an alternative energy source, and help maintain the redox balance. These metabolites have been shown to be important in cancer metabolism in both a tumorigenic and tumor-suppressive way and changes in their levels may be detected as cancer biomarkers and be targets for therapy [20].

In the present study, we have characterized metabolite profiles in preoperative plasma from patients who underwent potentially curative resection for suspected periampullary cancer and investigated associations to clinicopathological variables.

## Methods

### Clinical cohort

The patients (*N* = 117) were admitted to Oslo University Hospital from October 2008 to December 2017 for pancreatic resection due to suspicion of periampullary cancer. Signs of metastases at the time of the preoperative consultation was an exclusion criterion. Six patients had received neoadjuvant chemotherapy prior to surgery due to locally advanced disease. All patients underwent surgery with curative intent. Plasma and tissue samples were collected prior to and post-surgery, respectively. Pathology evaluation of the resected tissues was conducted, and a definite diagnosis was independently set and verified by two experienced pathologists. Tumors were classified following the TNM classification of malignant tumors available at the time of evaluation [21–23]. The collection of data and the analyses were performed in a retrospective manner.

### Ethical considerations and follow-up

All patients in the present study were included in the specific biobank at Oslo University Hospital entitled “Thematic Research Area Pancreatic Cancer”. The biobank was approved by the Regional Ethics Committee with reference REK 2015/738. The patient’s informed signed consent covers collection and analyses of biological material in addition to clinical information. The last date of follow-up was 27^th^ of January 2020 and included clinical status, radiological findings, and date of possible relapse of disease and death.

### Blood plasma – collection, preparation and storage

Venous blood samples (Ethylenediaminetetraacetic acid/EDTA) were collected from the patients during the clinical work-up, typically within two weeks prior to surgery. We do not have precise dietary plans of the patients in this cohort. To investigate if there were metabolite differences between fasting and non-fasting patients, we compared metabolite levels in the 64 patients with blood sample drawn the day of surgery (group A) with the 53 remaining patients (group B). We expect the patients in group A to be fasting and most of the patients in group B to be non-fasting at the time the blood sample was drawn. In the benign group (27 in group A, 18 in group B), none of the amino acids were found to be significantly different (Wilcoxon’s rank sum test). In the malignant group (37 in group A, 35 in group B), Leucine and Phenylalanine were found to be significantly different (FDR < 0.05) after multiple test-correction (Wilcoxon’s rank sum test). Both amino acids had lower medians in the non-fasting patients; the same trend was found in the benign patients (although not significant). In the aftermath, seven blood samples were discovered to have a collection date post-surgery. We did not detect significant differences in metabolite levels between the samples collected prior to and post-surgery. Hence, the samples collected after surgery were included in the analyses.

Plasma was isolated shortly after the blood sampling for the samples collected between 2008 and 2010. During the sampling period between 2011 and 2017, the blood samples were stored overnight at 4 °C before plasma isolation. Regardless of sampling period, all samples were centrifuged at 2272 g (3360 rpm) for 12 min at 4 °C and the plasma was stored at -80 °C until the present analysis.

The potential difference introduced by the two collection methods was investigated in the malignant and benign samples separately. In the benign group, Aspartic acid was the only amino acids that was significantly different between the two collection methods (*P* < 0.05, Wilcoxon’s Rank Sum Test), (Additional file 1). In the malignant group, ten of the metabolites were significantly different (*P* < 0.05, Wilcoxon’s Rank Sum Test) when comparing the two collection methods (Additional file 1). Six of these have been reported as stable as long as handled cold, and for Tryptophane even at room temperature [24, 25]. The metabolites were similarly expressed and the same differences in metabolite levels were found when comparing the benign to the malignant samples in the two collection groups separately. Principal component analysis (PCA) of all samples did not reveal a clear separation based on sampling handling time (Additional file 2). If present, an effect of the sample handling on the significant differences in the metabolite profiles between the benign and the malignant samples is therefore likely to be weak.

### Metabolite profiling

The plasma concentrations of amino acids and derivatives were measured by liquid chromatography–tandem mass spectrometry (LC-MS/MS) as described in Turner C. et al. [26]. Coefficient of variation for the analytes ranged from 4.6 to 9.2%. The 28 metabolites analyzed included twenty proteinogenic non-essential and essential amino acids, seven metabolites associated with amino acid metabolism that include the urea cycle associated metabolites Ornithine and Citrulline, the sulfur containing metabolites Cystathionine, Homocysteine, Taurine and Glutathione and the amino acid derivate Homoalanine also called α-Aminobutyric acid. In addition, because systemic disease is generally associated with muscle catabolism, Creatinine was a metabolite of choice (Fig. 2).

### Integration and normalization of data

The samples (*N* = 117) were analyzed by LC-MS/MS in two sets with nine overlapping samples. The nine overlapping samples were randomly chosen from the first set. The samples were from seven patients with PDAC, one patient with ampullary cancer, and one patient with cholangiocarcinoma. See Additional file 3 for detailed patient characteristics of the overlapping samples. The overlapping samples were used to normalize the two data sets (*N* = 9). The mean of each of the metabolites for each set for the nine overlapping samples were calculated. The metabolite values from each set were then divided by the mean value of the respective metabolite from the corresponding set of overlapping samples. For the overlapping samples, the mean values of the metabolites were used as one data point per metabolite. The integrated data set was log2-transformed and mean centered.

### Survival analysis

OS was calculated from the date of surgery to the date of death by any cause. Relapse-free survival (RFS) was defined as the time from the date of surgery to the date of radiologically confirmed relapse (local/metastatic disease). In a few cases where radiological imaging was not performed, the date was set to clinically and/or biochemically confirmed relapse. Associations between OS, clinical variables, and metabolite levels were examined using Cox regression models and the Kaplan–Meier estimator.

Six patients that received neoadjuvant chemotherapy, one patient with metastatic disease at the time of diagnosis and one patient with non-standard treatment regime were excluded from the survival analyses.

### Statistical methods

All statistical analyses were performed in R version 4.2.2 [27]. Differences in metabolite profiles between groups of samples were tested with Wilcoxon Rank Sum test and False Discovery Rate (FDR) values were reported. Plots were made using packages “ggfortify” [28], “ComplexHeatmap” [29], “corrplot” [30], “rms”, “survminer”, “reshape2” [31], and “pROC” [32]. PCA was used to detect variation between groups of data. Consensus clustering was performed for all samples on all metabolites to identify robust patterns. Briefly, consensus clustering consisted of 1000 iterations across k-values (cluster counts) ranging from 2–5 [33]. Each iteration of clustering involved resampling of 80% of features and items. Evaluation of the consensus matrices and cumulative distribution function (CDF) plots suggested that k = 2 clusters are optimal or close to optimal (Additional file 4). Subsequently, a hierarchical clustering of the consensus matrix with Pearson correlation distance and complete linkage was performed. Associations between metabolites and clinicopathological variables to OS were investigated by multiple Cox regression with a stepwise regression algorithm with backwards elimination. The backwards elimination algorithm started with a regression model with all variables included. The variable with the largest *p*-value was identified. If the *p*-value was larger than 0.05, the variable was excluded. The regression model was refitted, and the above steps were repeated until all remaining variables had a *p*-value less than 0.05.

## Results

### Demographic characteristics of the patients

In total, 72 patients with periampullary cancer and 45 patients with benign or precancerous periampullary disease were included in the present study. Sixty-four of the patients were men (57.3%), the median age was 67 years (range: 31–88 years), 26 had diabetes (22.2%) and 48 (43.2%, NA = 6) filled the criteria for moderate (20.7%, *N* = 23) or severe (22.5%, *N* = 25) malnutrition at the time of diagnosis. Clinical features, treatment, diagnosis, and histopathological results are detailed in Table 1 and Additional file 5.

**Table 1:** Clinical characteristics for the benign and malignant patients in the study cohort

Variable	Malignant disease (*N* = 72)		Benign disease (*N* = 45)	
Age, years (median, (range))	66.9 (34.7–87.6)		66.9 (32.0–80.9)	
Gender (N, (%))				
Male	38 (52.8%)		26 (57.8%)	
Female	34 (47.2%)		19 (42.2%)	
BMI (median, (range))	23.8 (14.5–43.1)	(NA = 1)	25.2 (18.1–35.4)	(NA = 1)
Nutritional status (N, (%))				
No malnutrition	27 (39.1%)	(NA = 3)	36 (85.7%)	(NA = 3)
Moderate	19 (27.5%)		4 (9.5%)	
Severe	23 (33.3%)		2 (4.8%)	
Diabetes mellitus (N, (%))				
Yes	15 (20.8%)		11 (24.4%)	
No	57 (79.2%)		34 (75.6%)	
Blood type (N, (%))				
A	42 (58.3%)		24 (53.3%)	
B	6 (8.3%)		2 (4.4%)	
O	23 (31.9%)		15 (33.3%)	
AB	1 (1.4%)		4 (8.9%)	
CA19-9 (median, (range))	184.5 (4–8403)	(NA = 16)	15 (4–61)	(NA = 16)
Neoadjuvant chemotherapy (N, (%))	6 (8.3%)		0 (0%)	
Surgery (N, (%))				
Whipple	23 (31.9%)		7 (15.6%)	
Pylorus preserving Whipple	43 (59.7%)		28 (62.2%)	
Total pancreatectomy^a^	5 (6.9%)		-	
Distal pancreatic resection	1 (1.4%)		9 (20.0%)	
Cholecystectomy	-		1 (2.2%)	
Diagnosis (N, (%))				
PDAC	59 (81.9%)		IPMN 15 (33.3%)	
Ampullary cancer	3 (4.2%)		Pancreatitis 12 (26.7%)	
Cholangiocarcinoma	5 (6.9%)		Other entities^b^ 18 (40.0%)	
Mucinous/IPMN associated carcinoma	4 (5.6%)			
Malignancy with uncertain origin	1 (1.4%)			
Recurrence status at 2-year follow-up (N, (%))				
Yes	56 (83.6%)	(NA = 5)	0 (0%)^c^	
No	11 (16.4%)		45 (100%)	
Survival status (last date of follow-up), (N, (%))				
Alive	8 (11.1%)		40 (88.9%)	
Dead	64 (88.9%)		5 (11.1%)	
Overall survival in months (median, (range))	19.7 (1.2–114.8)		50.6 (22.9–135.7)	

Study population *N* = 117

Total range or percentage in the parentheses

*BMI* Body mass index [34], *CA19-9* Cancer antigen 19–9

^a^Total pancreatectomy includes total pancreatectomy and total pancreatoduodenectomy

^b^Other entities include all other benign entities than Intraductal Papillary Mucinous Neoplasm (IPMN) and pancreatitis

^c^No patient diagnosed with benign disease had known development of cancer in the periampullary region by the time of last follow-up

The malignant tumors consisted of PDAC (82%) and a few cases of cholangiocarcinoma, ampullary cancer, and mucinous/intraductal papillary mucinous neoplasm (IPMN)-associated carcinoma. The patients with benign disease were diagnosed with pancreatitis, IPMN, and other benign entities in the periampullary region (Table 1). The treatment and follow-up after surgery depended on the diagnosis. Additional descriptions, including tumor size, resection margins, and histological subgroups were also dependent on the diagnosis and detailed in Additional file 5.

Of the cancer patients, 51.5% (35/68, NA = 4) had confirmed relapse (local and/or distant) of the disease within one year and 83.6% (56/67, NA = 5) within two years after surgery. The correlation between RFS and OS was high (0.94, *P* < 2.2*10^–16^, Pearson correlation), death following shortly after relapse (median 6.9 months, range: 0.8–37.4 months). The median OS of the cancer group was 19.7 months (range: 1.2–114.8 months). Postoperative 30-day mortality was zero. The cancer group included one patient with metastasis to the liver discovered during surgery and one patient with a non-standard treatment regime with an OS of 9.1 and 114.8 months, respectively. At the last update of clinical data, 27th of January 2020, eight of the 72 (11.1%) cancer patients were alive with a median follow-up of 57.6 months (range: 39.9–103.8 months), seven of them without known relapse of the disease.

In contrast, none of the patients with benign disease had developed cancer in the periampullary region in the follow-up period and 40 of the 45 (88.9%) patients with benign or precancerous disease were alive at the last date of follow-up. The five deceased patients died of other causes than malignant disease in the periampullary region.

### Plasma metabolite profiles are different between patients with cancer and benign disease

**Fig. 1:**
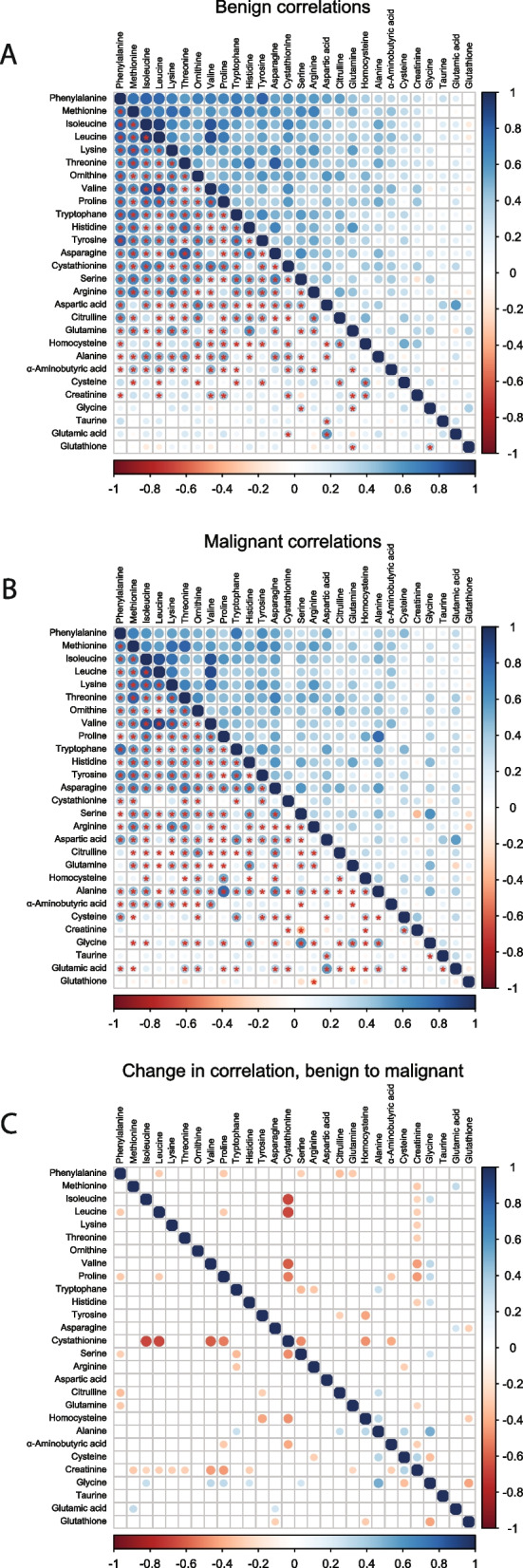
Plasma metabolite correlation plots based on Spearman correlation. **A** Correlation of the metabolites in the benign samples. **B** Correlation of the metabolites in the malignant samples. **C** The change in correlation comparing the benign to the malignant correlations. The color of the dot represents the direction of correlation on a continuous scale from blue (positive) to red (negative) correlation. The size of the dot indicates the strength of the correlation and the star in the lower quadrant indicates if the correlation is significant (*P* < 0.05). In panel **C**, only changes in correlation > 0.25 are visualized

In general, lower positive correlations were observed within the malignant samples in comparison to within the benign ones (Fig. 1A-B). In the benign samples, Cystathionine was positively correlated with Proline (Spearman correlation coefficient (ρ) 0.584, *P*-value < 0.001) and the Branched-chain amino acids (BCAA) Valine, Isoleucine, and Leucine (ρ 0.648, 0.683, and 0.616, respectively, *P*-value < 0.001). In the malignant samples, Glutamine was negatively correlated to Glutamic acid (ρ -0.254, *P*-value < 0.05), Glutathione was negatively correlated to Arginine (ρ -0.262, *P*-value < 0.05), and Serine was negatively correlated to Creatinine (ρ -0.358, *P*-value < 0.005). In addition, a significant positive correlation was observed between Glycine and Alanine (ρ 0.490, *P*-value < 0.001) in the malignant samples. The changes in correlations from the benign to the malignant samples are illustrated in Fig. 1C.

**Fig. 2:**
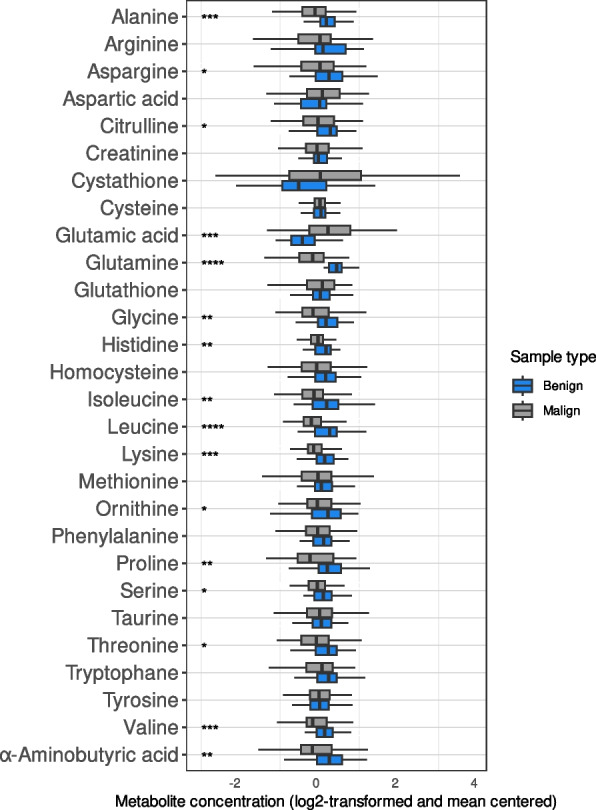
Boxplot illustrating the difference in metabolite profiles between samples from patients with benign pancreatic disease (blue, *N* = 45) and patients with periampullary malignancy (grey, *N* = 72; including 6 patients with neoadjuvant treatment). On the x-axis, the metabolite concentration (log2-transformed and mean centered). Wilcoxon’s Rank Sum Test was used to calculate the *p*-values. Multiple testing was performed by the Benjamini-Hochberg method (FDR). Significant difference in expression between the malignant and benign group for each metabolite is marked with stars; *: FDR < = 0.05, **: FDR < = 0.01, ***: FDR < = 0.001, ****: FDR < = 0.0001

The distribution of each metabolite varied with Cysteine and Cystathionine having the narrowest and the widest range, respectively (Fig. 2). The levels of more than half of the metabolites were significantly higher in the benign compared to the malignant samples. Glutamic acid was an exception with a significantly lower level in the benign samples (Fig. 2).

**Fig. 3:**
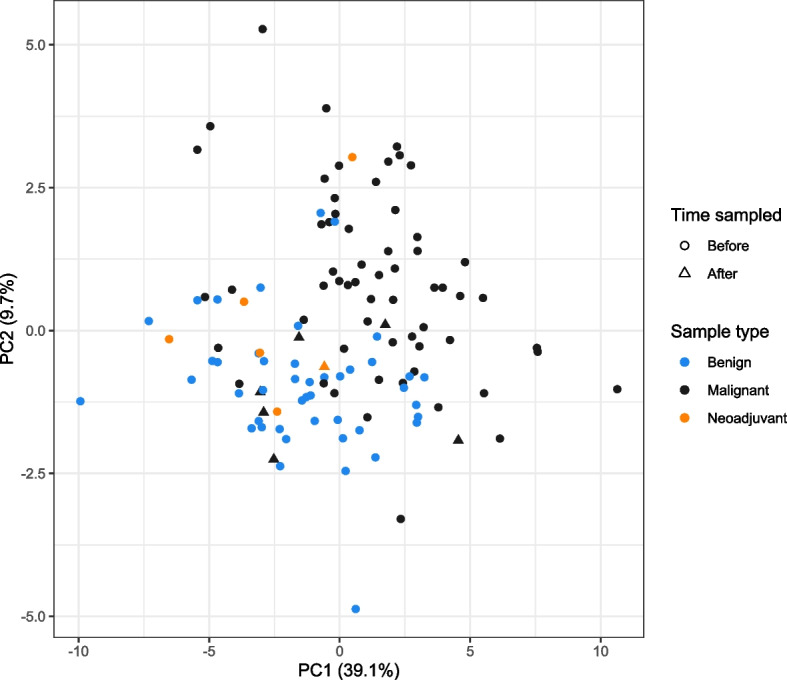
PCA plot of the metabolite profiles of patients with benign and malignant periampullary disease. Each dot represents a sample. Each color represents a given type of sample: Black = malignant (*N* = 60), Blue = benign (*N* = 45), Orange = malignant sample with neoadjuvant treatment (*N* = 6). The shape of the dot represents if the sample was collected before or after surgery: Circle = Before, Triangle = After. One sample had both neoadjuvant chemotherapy and a post-surgery sampling date

In the PCA plot, the patients with cancer and the patients with benign disease were partially separated from each other in the space of the first two principal components (black and blue dots in Fig. 3). The PCA plot further illustrates that the vast majority of the postoperative samples and the samples from patients with neoadjuvant chemotherapy grouped with the benign samples (Fig. 3). In contrast to no significant differences between the metabolite levels in the pre- and postoperative samples, six metabolites were significantly different when comparing the metabolite levels in the 66 patients without and the six patients with neoadjuvant treatment (Additional files 6 and 7). It should be noted that five out of these six metabolites were significantly different when comparing the malignant to the benign samples, and that the metabolite levels in the neoadjuvant samples were similar to the levels in the benign samples when compared to the malignant samples (Fig. 2, Additional file 6). The differences between the IPMN (premalignant) samples and the malignant and benign samples were investigated and presented in a PCA plot which illustrates the evenly distributed IPMN among the other benign samples (Additional file 8).

**Fig. 4:**
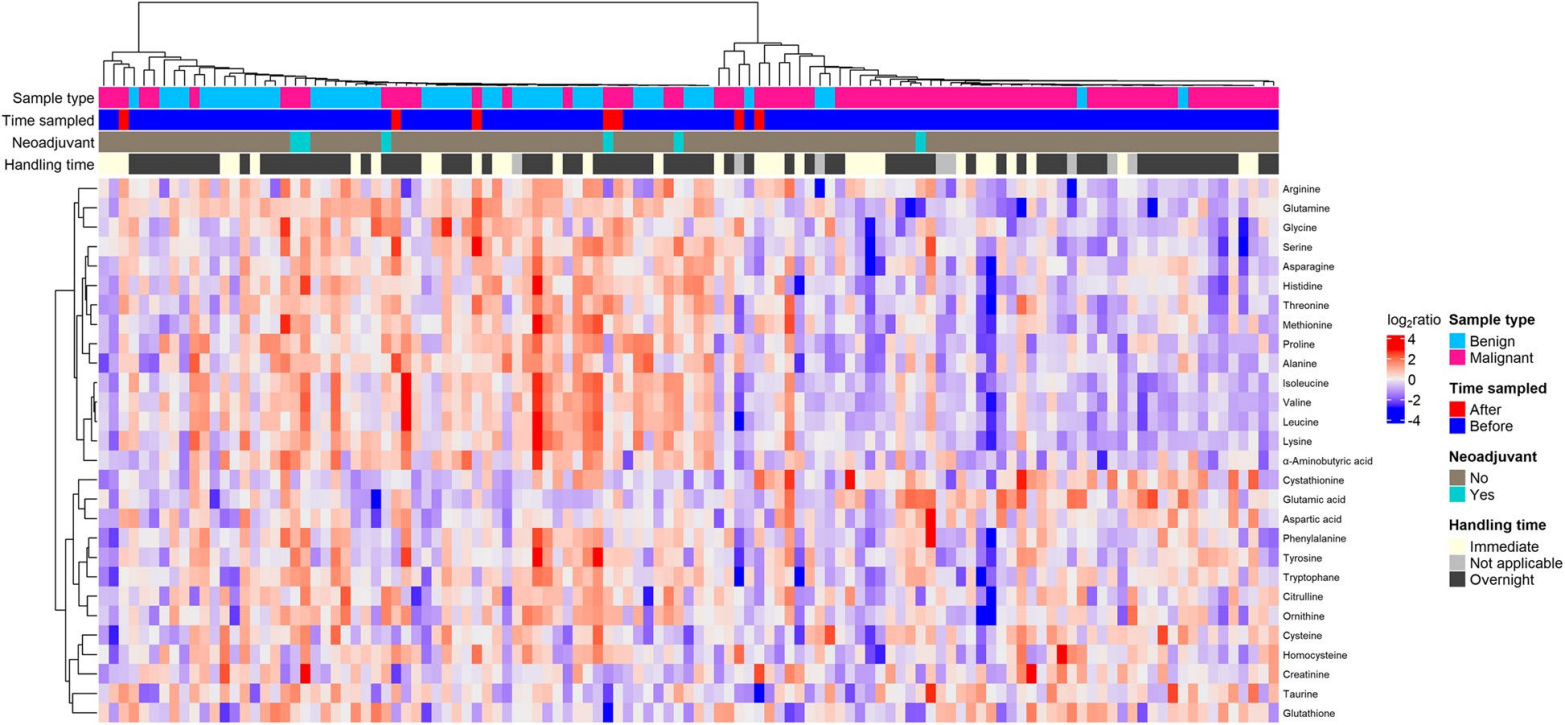
Consensus clustered heatmap of 117 patients and 28 metabolites. Four bars beneath the cluster tree illustrate if the given sample is benign (light blue) or malignant (pink), sampled before (dark blue) or after (red) surgery, if the sampled patient had received neoadjuvant treatment (turquoise) or not (grey), and if plasma was isolated from blood immediately after sampling or if the blood was stored overnight before pre-processing

Next, we performed consensus clustering of all the samples. Again, the patients were stratified into two clusters (Fig. 4), with a significant association of benign and malignant samples to each cluster (*P* = 1.007*10^–10^, Fischer’s Exact Test). The cluster denoted “Benign cluster”, constituted 61 of the patients and included most of the patients with benign disease (*N* = 40). It also constituted 21 of the malignant cases. It should however be noted that five of these samples were from cancer patients that had blood drawn post-surgery and five samples were from patients that received neoadjuvant chemotherapy. The remaining patients (*N* = 51) diagnosed with malignant disease clustered separately in the “Malignant cluster”, together with five patients with benign disease.

The predictive value of the metabolite data was investigated by calculating the mean of each metabolite in the benign samples in “Benign cluster” and the malignant samples in “Malignant cluster”, respectively. The samples were classified as “benign-like” or “malignant-like” according to their overall correlation to the means in the two clusters. The metabolite-based classification of each sample compared to the true tissue type was illustrated with a Receiver operating characteristics (ROC) curve (area under the curve (AUC) = 0.85, Additional file 9).

### Plasma metabolites, clinical variables and overall survival

The essential amino acids Histidine, Lysine, Threonine as well as the BCAA family, Isoleucine, Leucine, Valine, were low in the patients with malign disease when compared to the patients with benign disease (Figs. 2 and 4). These metabolites together with reduced levels of the non-essential amino acids Alanine, Asparagine, Glutamine, Glycine, Proline and Serine, and the non-proteogenic α-aminobutyric acid, Ornithine and Citrulline, demonstrated that approximately 50% (15 of 28) of the metabolites analyzed were significantly reduced in the patients with malignant disease (Fig. 2). We further investigated if the metabolite levels were associated with clinicopathological features. In our cohort, pathological characteristics like tumor size, differentiation, and resection margins revealed no significant influence in OS (Additional file 10). Of the clinical variables, only the blood type O versus A (HR = 2.14, CI 1.2–3.8, *P* < 0.01) were associated with OS (Additional files 10, 11, 12 and 13). When examining the metabolite profiles of the cancer patients with blood types O and A, no significant difference was identified.

A hallmark of periampullary cancer and particularly PDAC is malnutrition, tissue wasting, and cachexia, the latter associated with severe metabolic changes [34]. The Norwegian national guidelines for prevention and treatment of malnutrition define moderate and severe malnutrition based on the degree of involuntary weight loss, BMI, and dietary changes [35]. The majority of the benign patients did not show indication of malnutrition in the clinical work-up (36 out of 42, NA = 3). Comparing the metabolite profiles of the benign patients with and without malnutrition, only the anabolic amino acid, Valine of the BCAA family, was significantly different (*P* = 0.02) and reduced in the malnourished group. On the other hand, the cancer patients separated into three groups where 27 patients were diagnosed with no malnutrition, 19 with moderate, and 23 patients with severe malnutrition (*N* = 69, 3 were not determined for nutritional status (NA = 3)). We did not detect any significant differences in metabolite levels in the three nutritional groups, and no significant association between nutritional status or BMI and OS, were identified (Additional files 14 and 15, respectively).

In addition, many patients developing periampullary cancer are diagnosed with late onset diabetes mellitus prior to or shortly after the cancer diagnosis [36–39]. In the present cohort the patients with diabetes mellitus were equally distributed among the benign and malignant patients (Table 1). No significant differences in metabolites between the patients with or without diabetes were identified, nor could we detect a significant association between conditions of diabetes and OS.

**Fig. 5:**
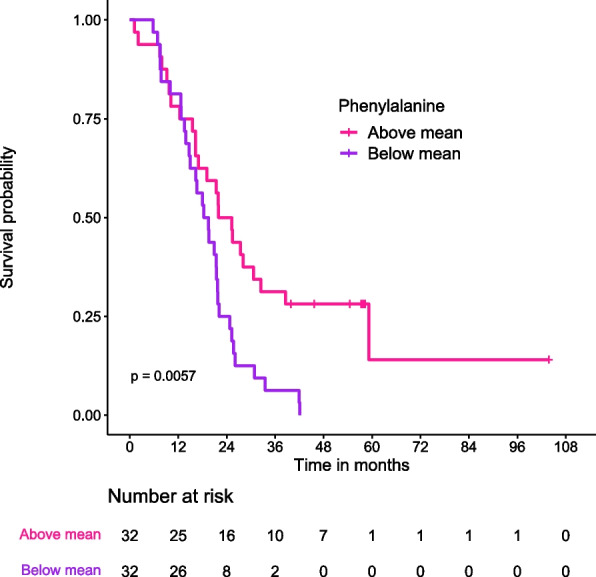
Kaplan-Meier curve illustrating overall survival of cancer patients with Phenylalanine levels above and below the mean. Patients that received neoadjuvant chemotherapy (*N* = 6), one patient with metastasis at the time of diagnosis and one patient with non-standard treatment regime were excluded

A single metabolite, Phenylalanine, which is solely proteogenic in muscle, was significantly associated with OS (Additional files 10, 13, and 16) in the malignant group. By dividing the cancer patients with Phenylalanine levels above and below the mean, the Kaplan-Meier curve illustrates the association between Phenylalanine levels and OS. After one year postoperatively the two survival curves split and around two years postoperatively 75% of the patients with low Phenylalanine levels were deceased compared to 50% of the patients with high levels as shown in Fig. 5.

## Discussion

In this study we analyzed the levels of 28 blood plasma amino acids and derivatives from 117 patients, all with suspicion of periampullary cancer. Of these patients, 45 were diagnosed with benign disease and 72 patients with periampullary cancer. Since PDAC constituted > 80% of the malignant tumors and no distinction could be made between the periampullary cancers preoperatively, we did not stratify within the malignant diagnosis. We found significantly different metabolite levels between the patients with benign and malignant disease in 16 of the 28 metabolites analyzed. Apart from Glutamic acid, we observed mainly lower metabolite levels in the malignant group.

The levels of the 3-carbon (3-C) non-essential amino acids Alanine, Glycine and Serine were all significantly reduced in plasma from patients with malignant compared to benign disease. In metabolically active cells, Serine and Glycine are crucial precursors for the synthesis of proteins, nucleic acids, and lipids [40–42]. Moreover, through the Glycine cleavage system, they refuel one-carbon (1-C) metabolism [43] crucial to protein-, nucleotide-, and glutathione synthesis in addition to being substrates for methylation reactions and epigenetic regulation [40]. In metabolically active cancer cells these processes are often dysregulated through oncogenic activity by enzymes such as Phosphoglycerate Dehydrogenase (PHGDH), Phosphoserine Aminotransferase 1 (PSAT-1) and Phosphoserine Phosphatase (PSPH) [40]. These enzymes are pivotal to Serine and Glycine metabolism, glycolytic activity and consequently Alanine-dependent Lactate production, a hallmark of the Warburg effect [44]. Our previous proteome analysis of PDAC tumor tissue revealed downregulation of ribosomal proteins and proteins responsible for endoplasmic processing [45]. Despite enigmatic, we speculate if down-regulated ribosomal activity and/or altered Warburg effect, are involved in aberrant regulation of 3-C amino acid levels in the patients with malignant disease.

Asparagine, with significantly lower levels in plasma of patient with periampullary cancer is known to be a key regulator of T cell activity. High Asparagine levels enhances endogenous tyrosine lymphocyte-specific protein tyrosine kinase (LCK) signaling to potentiate CD8+ T-cell activation and anti-tumor responses [46]. Low levels of Asparagine may reflect immune tolerance, further supporting tumor progression.

In contrast to the other metabolites analyzed here, circulating Glutamic acid was elevated in the patients with malignant disease. It has been demonstrated that high levels of extracellular Glutamate (the ionic form of Glutamic acid) may be due to a dysfunctional Cystine/Glutamate antiporter system. This antiporter system has been linked to aberrant stimulation of tumor-associated Glutamate receptors and promotion of tumor activity [47]. We speculate that reduced transport/cellular import of Glutamate may be associated with reduced levels of circulating Glutamine and Proline as these amino acids, which are tightly linked to cellular endogenous Glutamate synthesis [48], may be utilized as substrates to support low levels of endogenous Glutamate. To what extent this is the case needs further investigation.

The levels of the non-proteogenic amino acids Citrulline and Ornithine were significantly lower in the plasma of the cancer patients. Citrulline is synthesized from Ornithine in the intestine after which it may be released into circulation. This is in contrast to hepatic Citrulline, which is restricted from systemic circulating, when trapped in the Urea cycle together with Ornithine and Arginine. Based on this, we propose that low levels of Citrulline may reflect low intestinal synthesis. The cause of low Citrulline and Ornithine synthesis is enigmatic but may be linked to reduced levels of BCAA, which were also low in the patients with malignant disease. Biologically, a pivotal role of BCAA function is in maintaining an intestinal barrier, promoting intestinal development, enhancing enterocyte proliferation, and to promote increased intestinal absorption of amino acids and glucose [49]. Based on this, we speculate that low levels of BCAA may induce and hence act as measures and potential biomarkers for intestinal dysfunction in the patient group with malignant disease.

Forty-two of the patients with malignant disease suffered from moderate to severe malnutrition. It is well documented that patients with periampullary cancers suffer from muscular wasting and may develop cachexia [50]. BCAAs are pivotal in muscular energy metabolism and are considered anabolic for muscular protein synthesis [51]. We speculate that intestinal dysfunction and low nutritional status in the patients with malignant disease are symptoms linked to low levels of anabolic BCAA but also muscle wasting. The amino acid Phenylalanine is important for muscular protein synthesis but is not catabolized in muscle tissue [52, 53]. Despite that Phenylalanine levels were not different between the benign and malignant patients, and no significant association was found between nutritional status and Phenylalanine levels, elevated levels of circulating Phenylalanine was associated with increased OS in the patients with malignant disease. No literature was found on the association between levels of plasma Phenylalanine and OS in cancer patients and our cohort’s clinical data held insufficient in-depth information on muscle wasting and cachexia.

Consensus clustering based on the 28 metabolites divided the patients into two distinct clusters, consisting mainly of either benign or malignant disease, suggesting that the metabolite profiles can distinguish malignant and benign periampullary disease. We also observed that the periampullary cancer cases with neoadjuvant chemotherapy grouped with the samples from patients with benign disease. This imply that neoadjuvant treatment is likely to skew the metabolite profiles in the malignant patient group towards a benign profile. We can only speculate if the differences in metabolite profiles in neoadjuvant treated patients are due to a direct effect of the chemotherapy or by an indirect effect caused by shrinkage of the malignant tumor. By removing the six neoadjuvant samples from the malignant group, 21 of 28 metabolites were significantly different between the remaining malignant samples and the benign samples (Additional file 17). This further confirmed distinctly different metabolic profiles between patients with malignant and benign disease.

## Conclusions

In this study, approximately 50% of the plasma metabolites analyzed exhibited significant differences between patients with malignant and benign periampullary disease. Apart from Glutamic acid, the metabolites had lower levels in the malignant compared to the benign samples. Of the metabolites, only the muscular proteogenic amino acid Phenylalanine had a significant association to prognosis where higher Phenylalanine levels associated to better OS.

Taken together, we propose that the plasma metabolite profile has the potential to differentiate between benign and malignant periampullary disease preoperatively.

### Supplementary Information

**Additional file 1.** Differences in metabolite profiles ascribable to pre-processing methods. Boxplots illustrating differences in metabolite profiles between samples pre-processed shortly after collection and samples stored at 4 °C overnight before pre-processing from A: benign (Immediate *N* = 11, Overnight *N* = 32, NA = 2) and B: malignant samples (Immediate *N* = 22, Overnight *N* = 38, NA = 6), respectively. On the x-axis, the metabolite concentration (log2-transformed and mean centered). Samples from patients that received neoadjuvant chemotherapy are excluded (*N* = 6). Wilcoxon’s Rank Sum Test was used to calculate the *p*-values. The *p*-values were adjusted by the Benjamini-Hochberg method. Significant difference in metabolite levels based on handling is marked with stars; *: FDR-value <= 0.05, **: FDR-value <= 0.01, ***: FDR-value <= 0.001, ****: FDR-value <= 0.0001.

**Additional file 2.** PCA plot of the metabolite profiles of patients with benign and malignant periampullary disease illustrating the distribution of handling time. Each dot represents a sample. Each color represents a given type of sample: Black = malignant, Blue = benign, Orange = malignant sample with neoadjuvant treatment. The shape of the dot represents if the sample was pre-processed immediately after blood collection or after overnight storage: Circle = Immediate, Triangle = Overnight. Eight missing values for handling time.

**Additional file 3.** Clinical characteristics for the benign, malignant, and overlapping patients between the two LC-MS/MS analysis sets in the study cohort. Study population *N* = 117. Total range or percentage in the parentheses. *Total pancreatectomy includes total pancreatectomy and total pancreatoduodenectomy. **Other entities include all other benign entities than Intraductal Papillary Mucinous Neoplasm (IPMN) and pancreatitis. ***No patient diagnosed with benign disease had known development of cancer in the periampullary region by the time of last follow-up. **** The nine overlapping samples between the two LC-MS/MS runs used for normalization. BMI = body mass index [50]. CA19-9 = Cancer antigen 19-9.

**Additional file 4.** Consensus clustering of all samples and all metabolites using different k-values, A: k = 2, B: k = 3, C: k = 4, D: k = 5. E: Plot showing the cumulative distribution functions (CDF) of the consensus matrix for each k (indicated by colors), estimated by a histogram of 100 bins. F: Plot showing the relative change in area under the CDF curve comparing k and k−1. For k = 2, there is no k-1, and the total area under the curve rather than the relative increase is plotted [54].

**Additional file 5.** Histopathology characteristics for the patients with malignant periampullary disease. Study population *N* = 72. Total range or percentage in the parenthesis.

**Additional file 6.** Differences in metabolite profiles ascribable to neoadjuvant treatment in malignant and benign samples. Boxplot illustrating the difference in metabolic profiles between samples from patients with periampullary cancer with neoadjuvant treatment (*N* = 6, Orange), patients with treatment-naive periampullary cancer (*N* = 66, Grey) and patients with benign pancreatic disease (*N* = 45, Blue). On the x-axis, the metabolite concentration (log2-transformed and mean centered). An overview of the resulting FDR when testing the difference in metabolites between cancer patients with and without neoadjuvant treatment is shown in Additional file 7.

**Additional file 7.** Differences in metabolite profiles ascribable to neoadjuvant treatment in malignant and benign samples – FDR values. Table with metabolites and associated FDR q-values when comparing samples from patients receiving neoadjuvant chemotherapy (*N* = 6) and cancer patients with no chemotherapy prior to surgery (*N* = 66)”. Wilcoxon’s Rank Sum Test was used to calculate the p-values. The *p*-values were adjusted for multiple testing by the Benjamini-Hochberg method (FDR). FDR-values < 0.05 are in bold. See Additional file 6. FDR = False Discovery Rate.

**Additional file 8.** PCA plot of the metabolite profiles of patients with benign, premalignant (IPMN) and malignant periampullary disease. Each dot represents a sample. Each color represents a given type of sample: Black = malignant (*N* = 60), Blue = benign (*N* = 30), Purple = premalignant (IPMN, *N* = 15). IPMN = Intraductal papillary mucinous neoplasm.

**Additional file 9.** ROC curve – Patients with benign and malignant disease. ROC curve of the classification from the “Benign cluster” and the “Malignant cluster” in Fig. 4 on patients with benign and malignant disease (*N* = 117). ROC = receiver operating characteristic, AUC = area under the curve.

**Additional file 10.** Associations between clinical variables and metabolites and overall survival – Univariable Cox regression. Univariable Cox Regression model for patients with periampullary cancer (*N* = 64). Patients that received neoadjuvant chemotherapy (*N* = 6), one patient with metastasis at the time of diagnosis and one patient with non-standard treatment regime were excluded. *P*-values < 0.05 are in bold. CI = Confidence Interval.

**Additional file 11.** Blood type and overall survival of cancer patients. Kaplan-Meier curve illustrating overall survival of cancer patients with blood types A and O. Patients that received neoadjuvant chemotherapy (*N* = 6), one patient with metastasis at the time of diagnosis and one patient with non-standard treatment regime were excluded. Patients with blood types B and AB are not illustrated (*N* = 5 and 1, respectively).

**Additional file 12.** Associations between selected clinical variables and metabolites and overall survival in patients with periampullary cancer. Multivariable Cox Regression model for patients with periampullary cancer (*N* = 64) prior to stepwise regression algorithm with backwards elimination. Patients that received neoadjuvant chemotherapy (*N* = 6), one patient with metastasis at the time of diagnosis and one patient with non-standard treatment regime were excluded. *P*-values < 0.05 are in bold. CI = Confidence Interval.

**Additional file 13.** Associations between selected clinical variables and metabolites and overall survival – Multivariable Cox regression. Multivariable Cox Regression model with stepwise regression algorithm with backwards elimination for patients with periampullary cancer (*N* = 64). Selection of variables based on univariable Cox regression model (Additional file 10). Patients that received neoadjuvant chemotherapy (*N* = 6), one patient with metastasis at the time of diagnosis and one patient with non-standard treatment regime were excluded. *P*-values < 0.05 are in bold. CI = Confidence Interval.

**Additional file 14.** Nutritional status and overall survival of cancer patients. Kaplan-Meier curve illustrating overall survival in cancer patients with no, moderate, and severe malnutrition. Patients that received neoadjuvant chemotherapy (*N* = 6), one patient with metastasis at the time of diagnosis and one patient with non-standard treatment regime were excluded. NA = 3.

**Additional file 15.** BMI and overall survival of cancer patients. Kaplan-Meier curve illustrating overall survival in cancer patients with normal weight (BMI 18.5–25), overweight (BMI 25–30, and obesity (BMI > 30) according to WHO’s classification. Patients that received neoadjuvant chemotherapy (*N* = 6), one patient with metastasis at the time of diagnosis and one patient with non-standard treatment regime were excluded. Underweight patients (*N* = 3, BMI < 18.5) are not illustrated. NA = 1.

**Additional file 16.** Plasma Phenylalanine and overall survival of cancer patients. Scatter plot illustrating the association between levels of plasma Phenylalanine and overall survival in cancer patients. Each dot represents a sample. The line represents the direction of the correlation. Patients that received neoadjuvant chemotherapy (*N* = 6), one patient with metastasis at the time of diagnosis and one patient with non-standard treatment regime were excluded.

**Additional file 17.** Differences in metabolite profiles in patients with benign and malignant disease - FDR values. Table with metabolites and associated FDR q-values when comparing samples from patients with benign (*N* = 45) and malignant (*N* = 66) periampullary disease. Patients with neoadjuvant chemotherapy were excluded (*N* = 6). Wilcoxon’s Rank Sum Test was used to calculate the p-values. The p-values were adjusted for multiple testing by the Benjamini-Hochberg method (FDR). FDR-values < 0.05 are in bold. See Fig. 2 and Additional file 6. FDR = False Discovery Rate.

## Data Availability

The clinical data that support the findings of this study are located in controlled access data storage at Oslo University Hospital and are not openly available due to reasons of sensitivity. Data requests should be directed to the corresponding author.

## Abbreviations

AUC: Area under the curve
BCAA: Branched-chain amino acids
BMI: Body mass index
CA19-9: Cancer antigen 19-9
CDF: Cumulative distribution function
CI: Confidence Interval
FDR: False Discovery Rate
HR: Hazard ratio
IPMN: Intraductal papillary mucinous neoplasm
LC-MS/MS: Liquid chromatography–tandem mass spectrometry
OS: Overall survival
PCA: Principal component analysis
PDAC: Pancreatic ductal adenocarcinoma
RFS: Relapse-free survival
ROC: Receiver operating characteristic
1-C: 1-Carbon
3-C: 3-Carbon
