# Supplementary figures and images for: Preoperative profiles of plasma amino acids and derivatives distinguish periampullary cancer and benign disease
Source: BMC Cancer. 2024 May 3;24:555. doi: 10.1186/s12885-024-12320-8 (PMC11067218; doi:10.1186/s12885-024-12320-8)

A

Benign samples

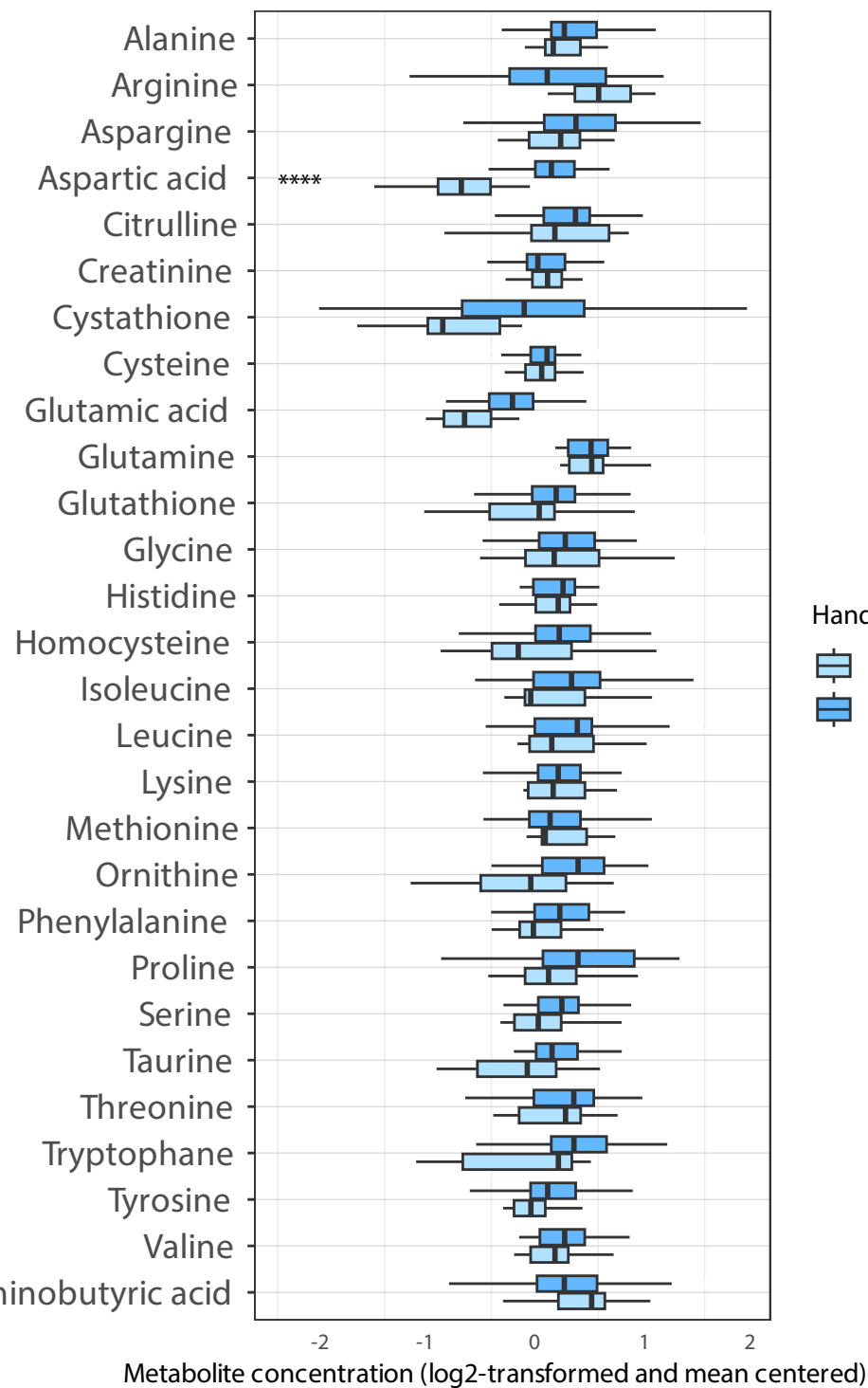

B

Malign samples

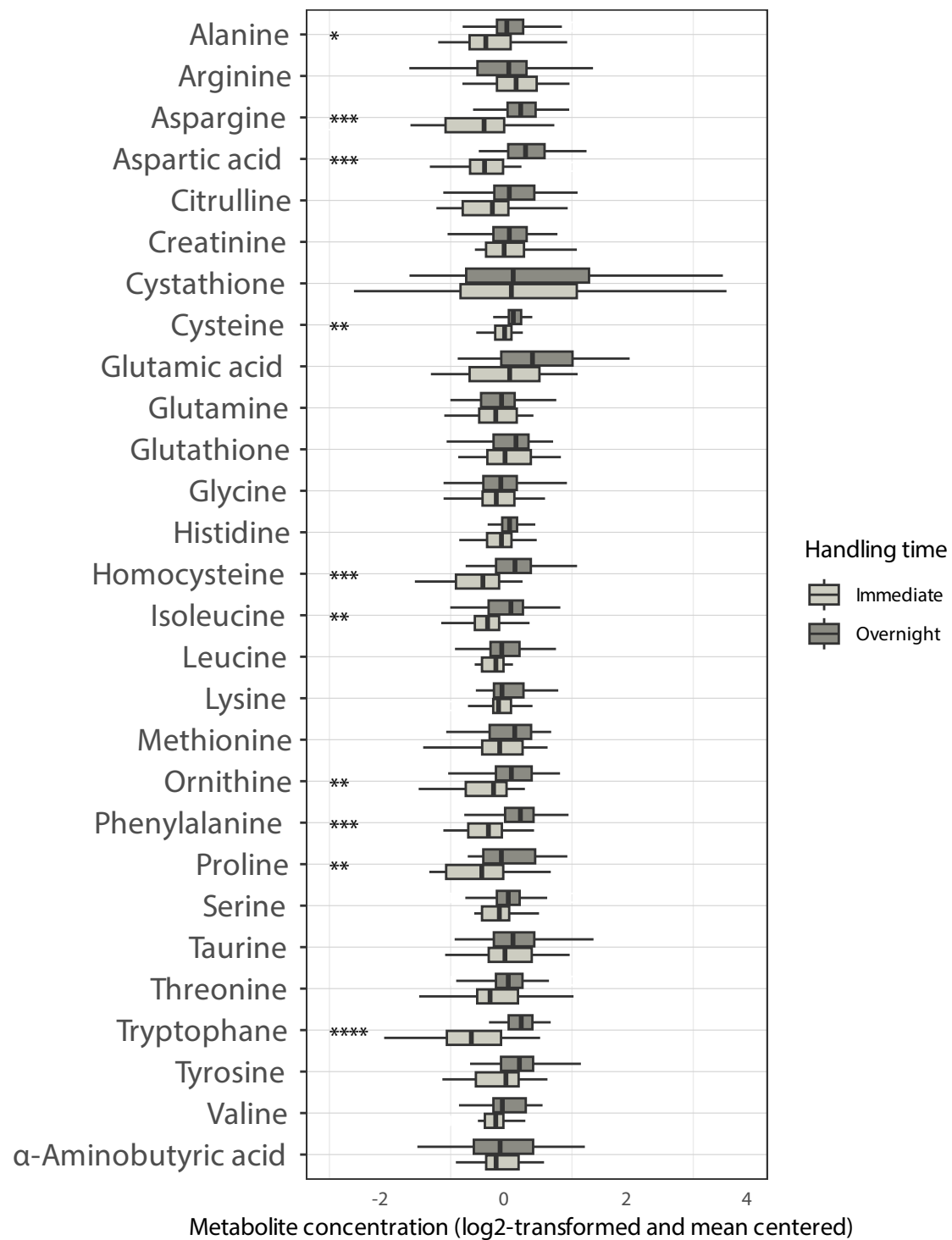

Supplement: Supplementary file 1 — Additional file 1. Differences in metabolite profiles ascribable to pre-processing methods. Boxplots illustrating differences in metabolite profiles between samples pre-processed shortly after collection and samples stored at 4 °C overnight before pre-processing from A: benign (Immediate N = 11, Overnight N = 32, NA = 2) and B: malignant samples (Immediate N = 22, Overnight N = 38, NA = 6), respectively. On the x-axis, the metabolite concentration (log2-transformed and mean centered). Samples from patients that received neoadjuvant chemotherapy are excluded (N = 6). Wilcoxon’s Rank Sum Test was used to calculate the p-values. The p-values were adjusted by the Benjamini-Hochberg method. Significant difference in metabolite levels based on handling is marked with stars; *: FDR-value <= 0.05, **: FDR-value <= 0.01, ***: FDR-value <= 0.001, ****: FDR-value <= 0.0001. [file 12885_2024_12320_MOESM1_ESM.pdf]

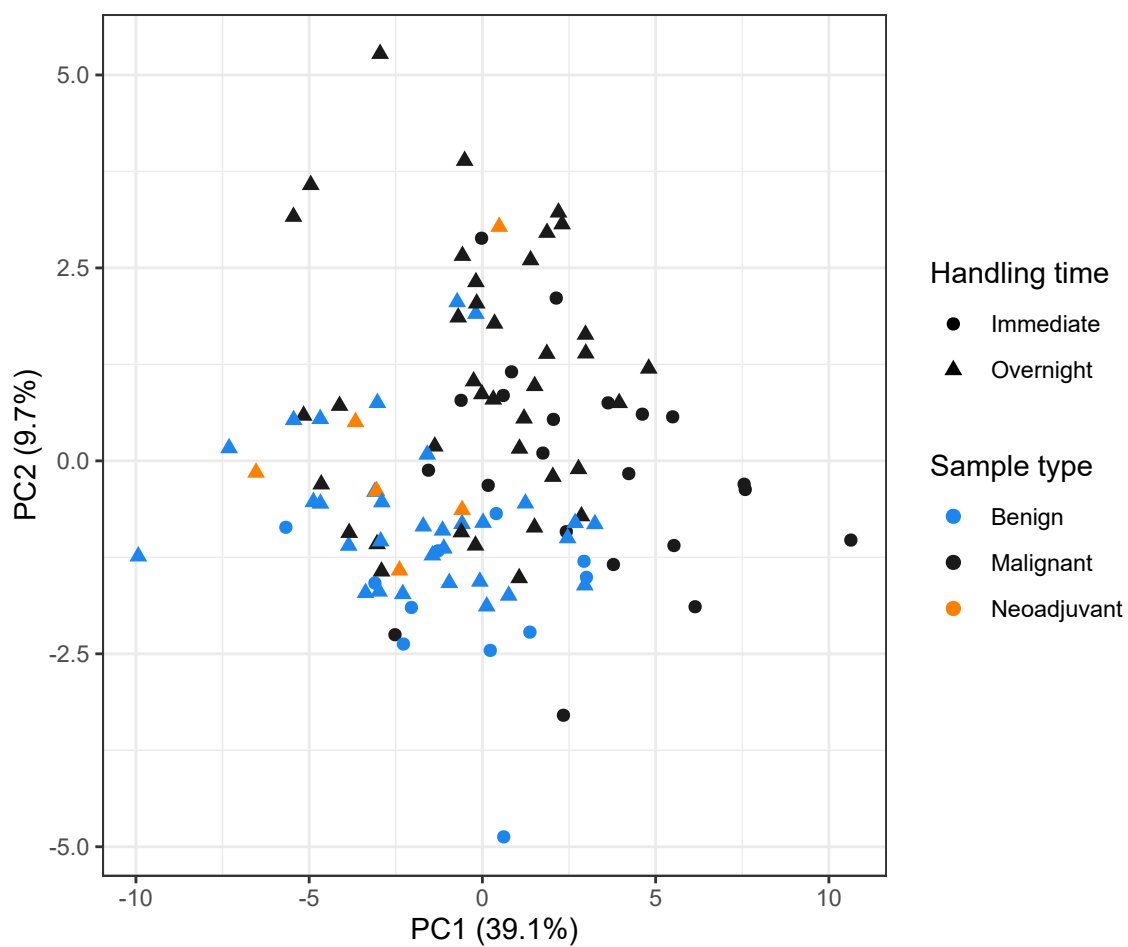

Supplement: Supplementary file 2 — Additional file 2. PCA plot of the metabolite profiles of patients with benign and malignant periampullary disease illustrating the distribution of handling time. Each dot represents a sample. Each color represents a given type of sample: Black = malignant, Blue = benign, Orange = malignant sample with neoadjuvant treatment. The shape of the dot represents if the sample was pre-processed immediately after blood collection or after overnight storage: Circle = Immediate, Triangle = Overnight. Eight missing values for handling time. [file 12885_2024_12320_MOESM2_ESM.pdf]

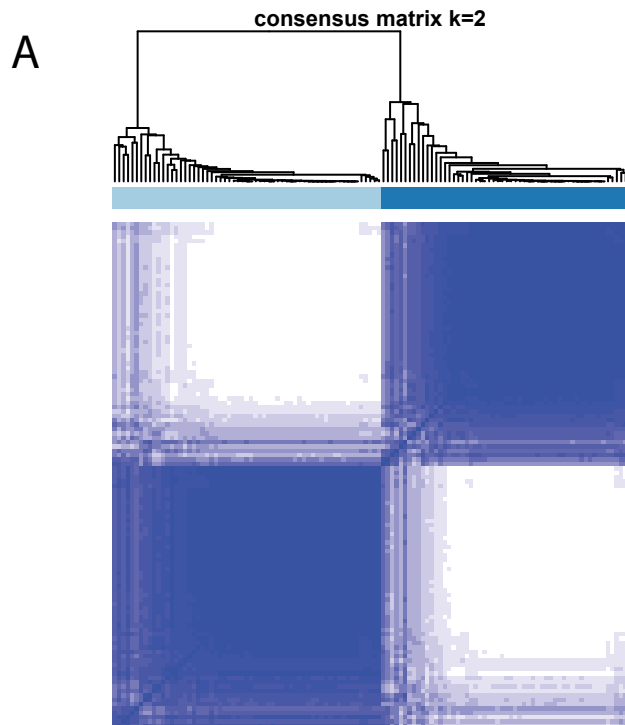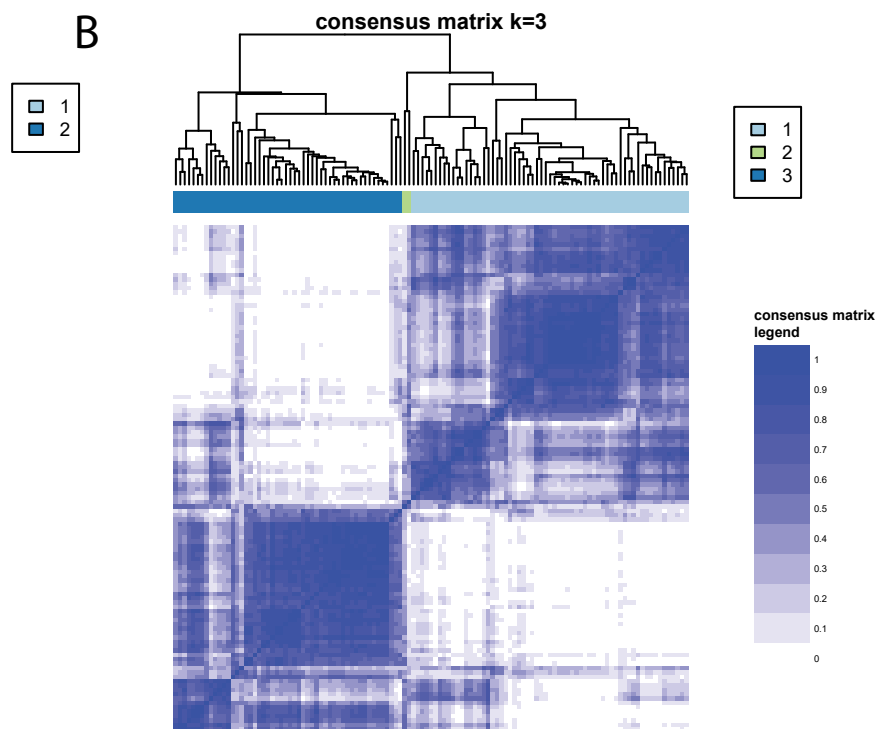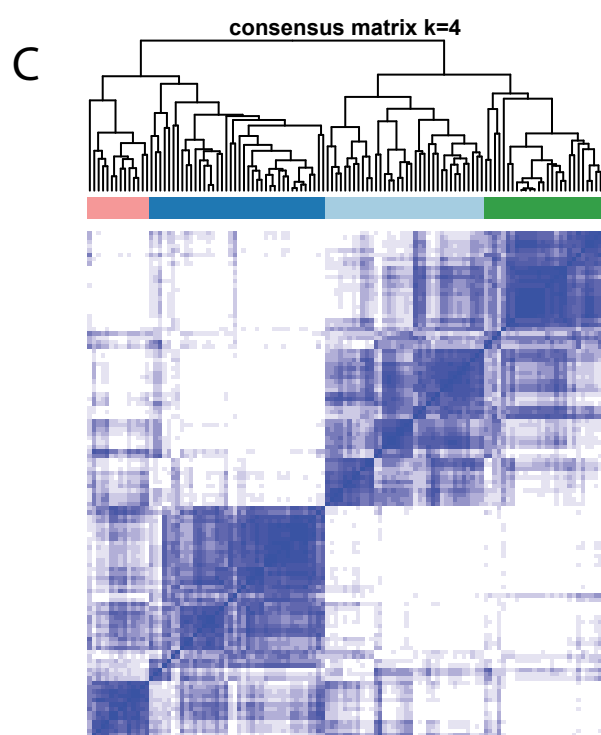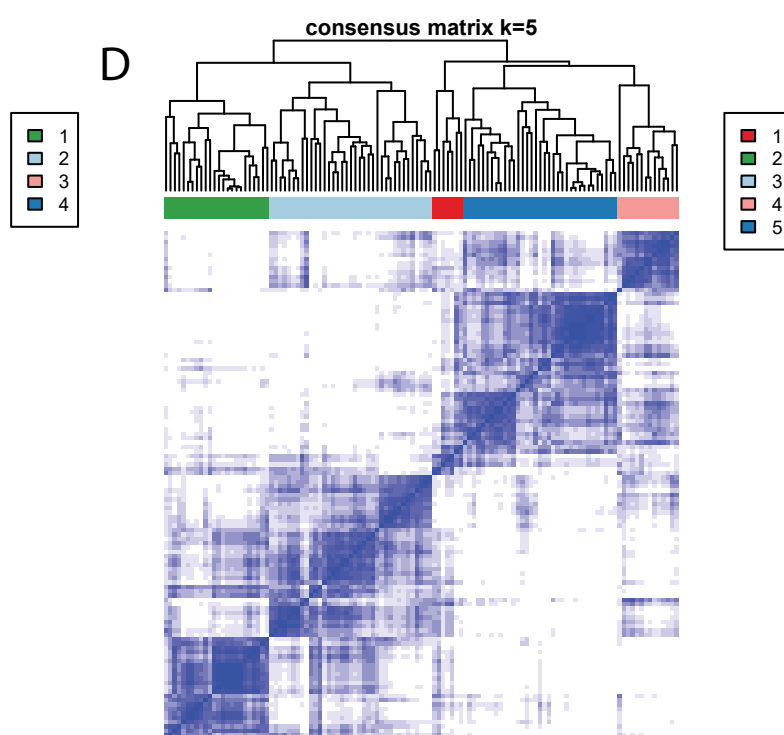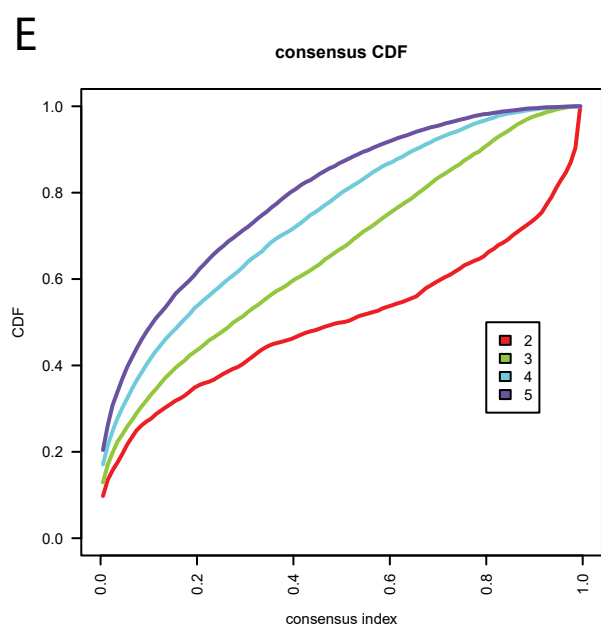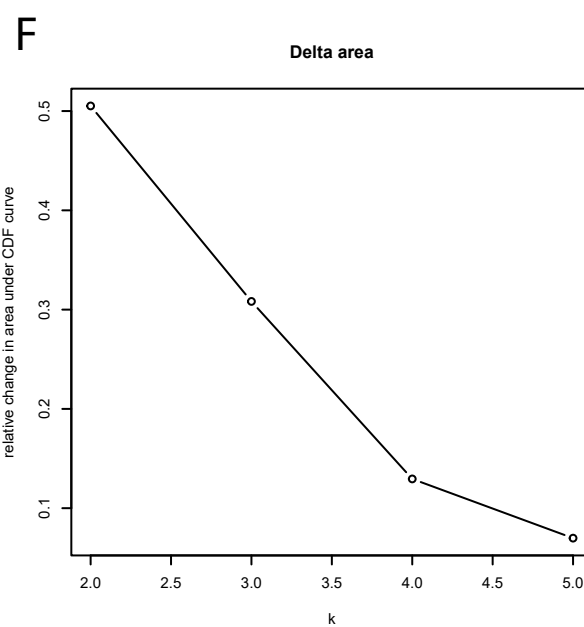

Supplement: Supplementary file 4 — Additional file 4. Consensus clustering of all samples and all metabolites using different k-values, A: k = 2, B: k = 3, C: k = 4, D: k = 5. E: Plot showing the cumulative distribution functions (CDF) of the consensus matrix for each k (indicated by colors), estimated by a histogram of 100 bins. F: Plot showing the relative change in area under the CDF curve comparing k and k−1. For k = 2, there is no k-1, and the total area under the curve rather than the relative increase is plotted [54]. [file 12885_2024_12320_MOESM4_ESM.pdf]

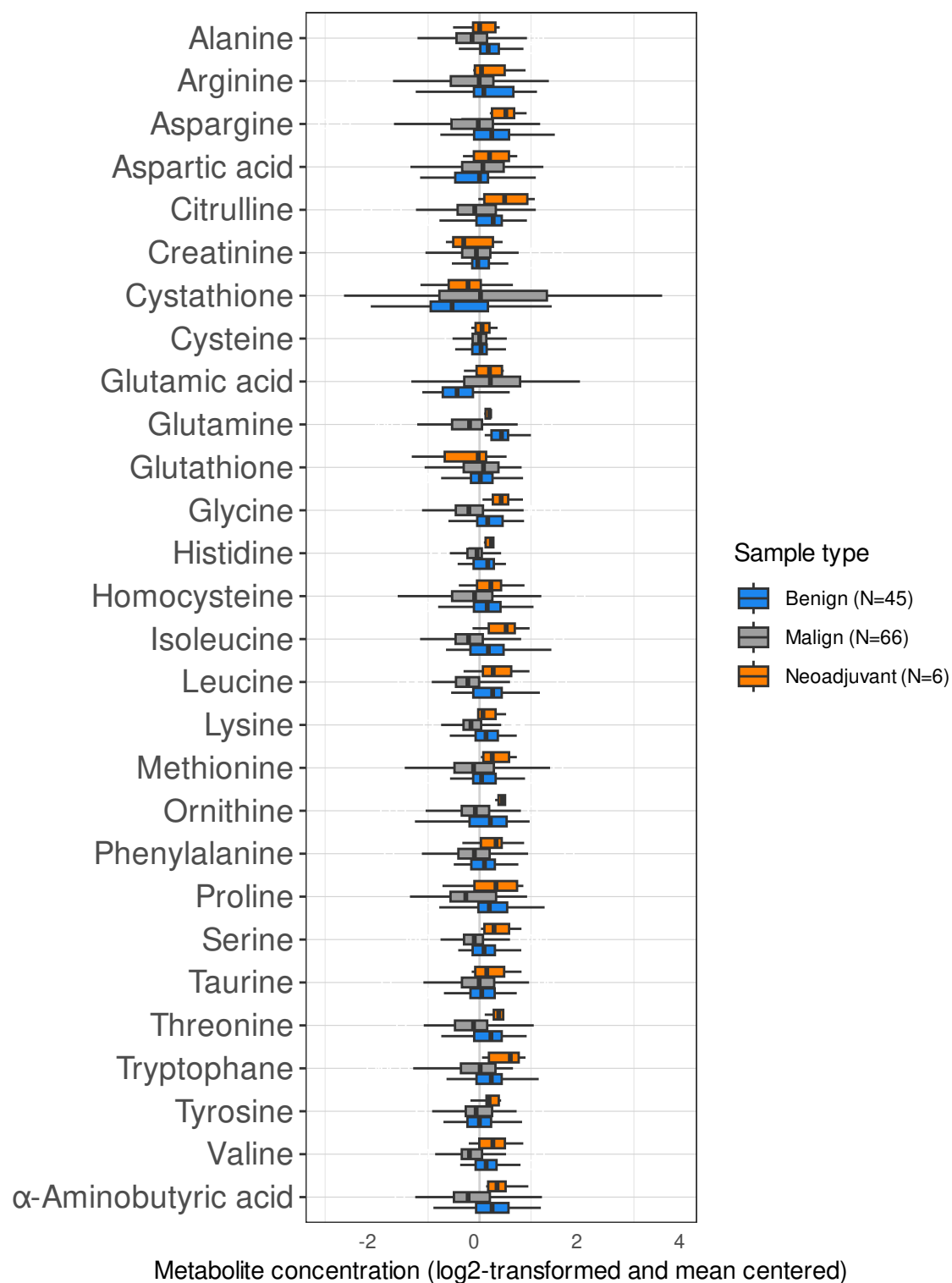

Supplement: Supplementary file 6 — Additional file 6. Differences in metabolite profiles ascribable to neoadjuvant treatment in malignant and benign samples. Boxplot illustrating the difference in metabolic profiles between samples from patients with periampullary cancer with neoadjuvant treatment (N = 6, Orange), patients with treatment-naive periampullary cancer (N = 66, Grey) and patients with benign pancreatic disease (N = 45, Blue). On the x-axis, the metabolite concentration (log2-transformed and mean centered). An overview of the resulting FDR when testing the difference in metabolites between cancer patients with and without neoadjuvant treatment is shown in Additional file 7. [file 12885_2024_12320_MOESM6_ESM.pdf]

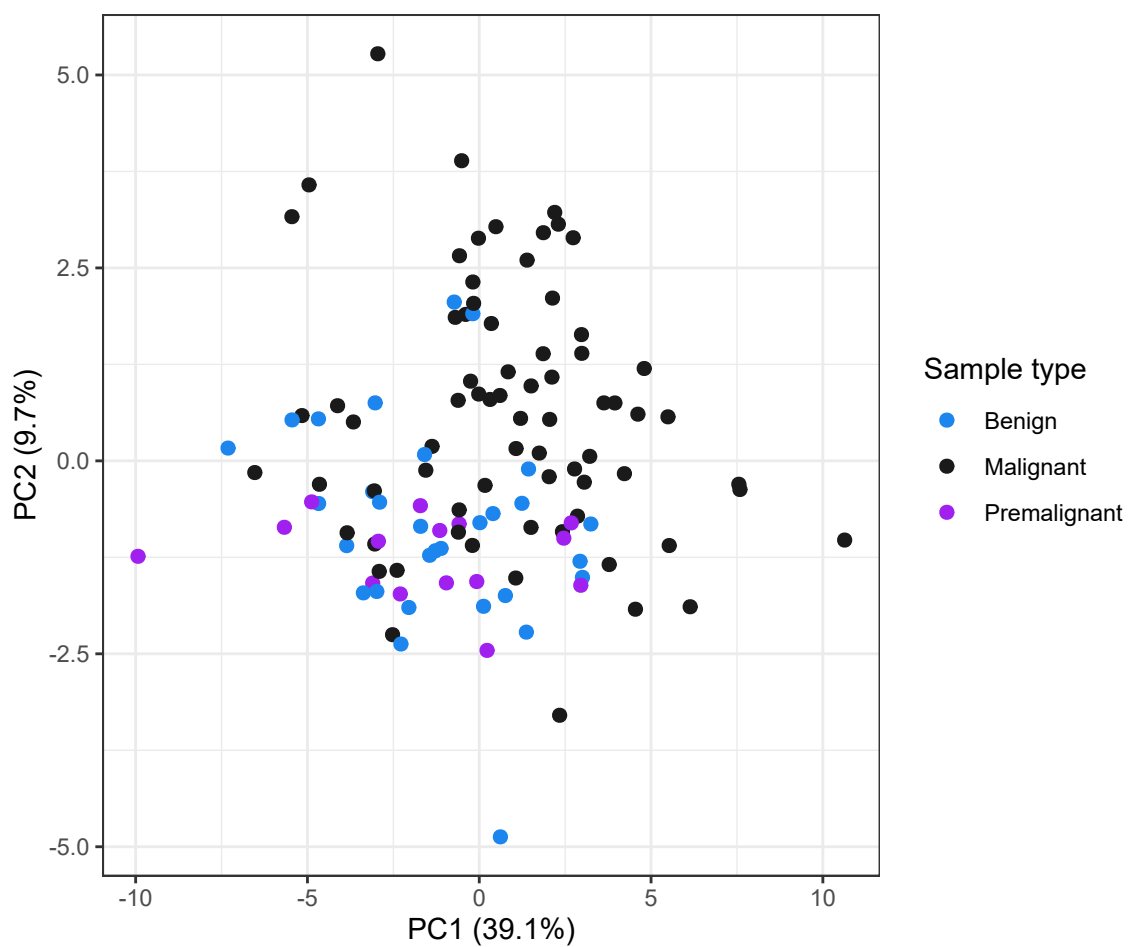

Supplement: Supplementary file 8 — Additional file 8. PCA plot of the metabolite profiles of patients with benign, premalignant (IPMN) and malignant periampullary disease. Each dot represents a sample. Each color represents a given type of sample: Black = malignant (N = 60), Blue = benign (N = 30), Purple = premalignant (IPMN, N = 15). IPMN = Intraductal papillary mucinous neoplasm. [file 12885_2024_12320_MOESM8_ESM.pdf]

ROC curve for all samples

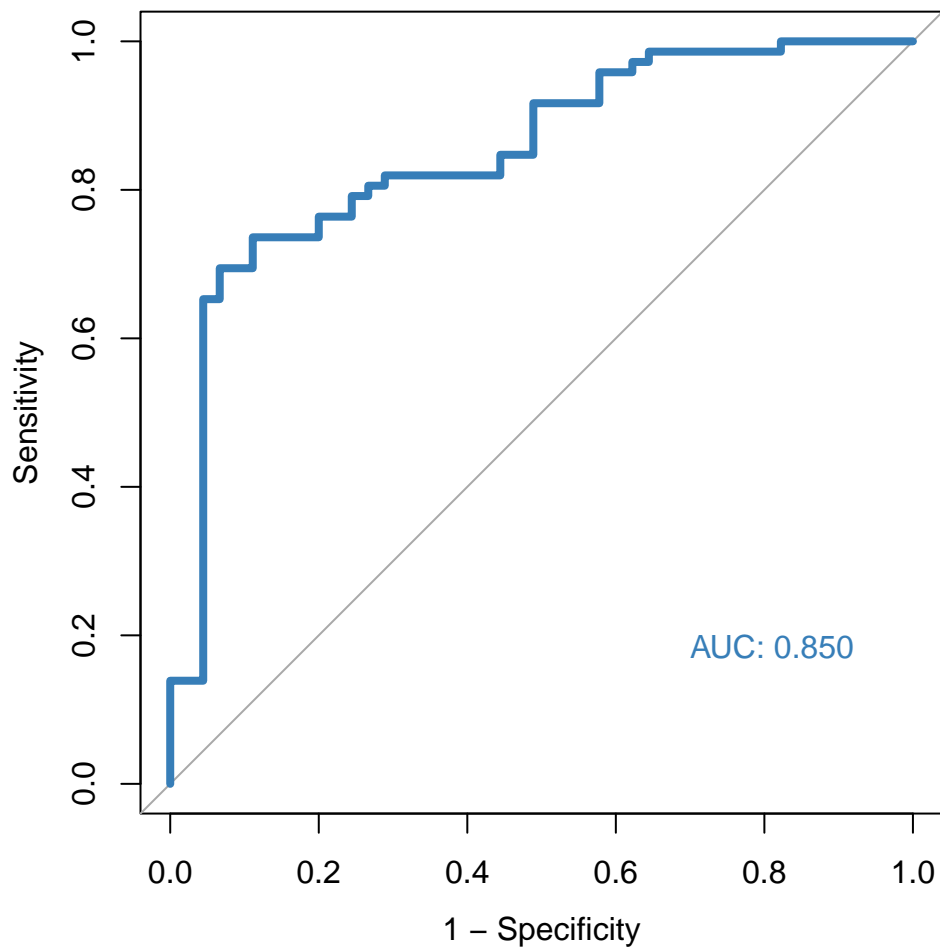

Supplement: Supplementary file 9 — Additional file 9. ROC curve – Patients with benign and malignant disease. ROC curve of the classification from the “Benign cluster” and the “Malignant cluster” in Fig. 4 on patients with benign and malignant disease (N = 117). ROC = receiver operating characteristic, AUC = area under the curve. [file 12885_2024_12320_MOESM9_ESM.pdf]

Survival probability

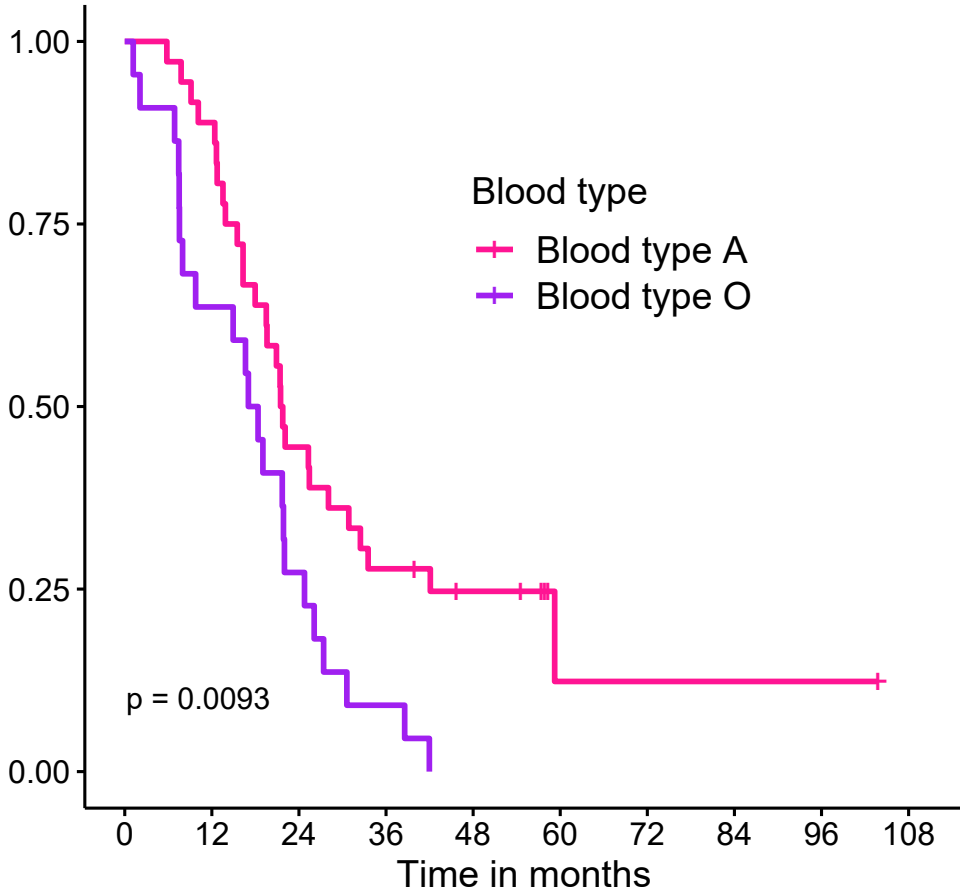

Number at risk

|              |    |    |    |    |   |   |   |   |   |   |
|--------------|----|----|----|----|---|---|---|---|---|---|
| Blood type A | 36 | 32 | 16 | 10 | 7 | 1 | 1 | 1 | 1 | 0 |
| Blood type O | 22 | 14 | 6  | 2  | 0 | 0 | 0 | 0 | 0 | 0 |

Supplement: Supplementary file 11 — Additional file 11. Blood type and overall survival of cancer patients. Kaplan-Meier curve illustrating overall survival of cancer patients with blood types A and O. Patients that received neoadjuvant chemotherapy (N = 6), one patient with metastasis at the time of diagnosis and one patient with non-standard treatment regime were excluded. Patients with blood types B and AB are not illustrated (N = 5 and 1, respectively). [file 12885_2024_12320_MOESM11_ESM.pdf]

Survival probability

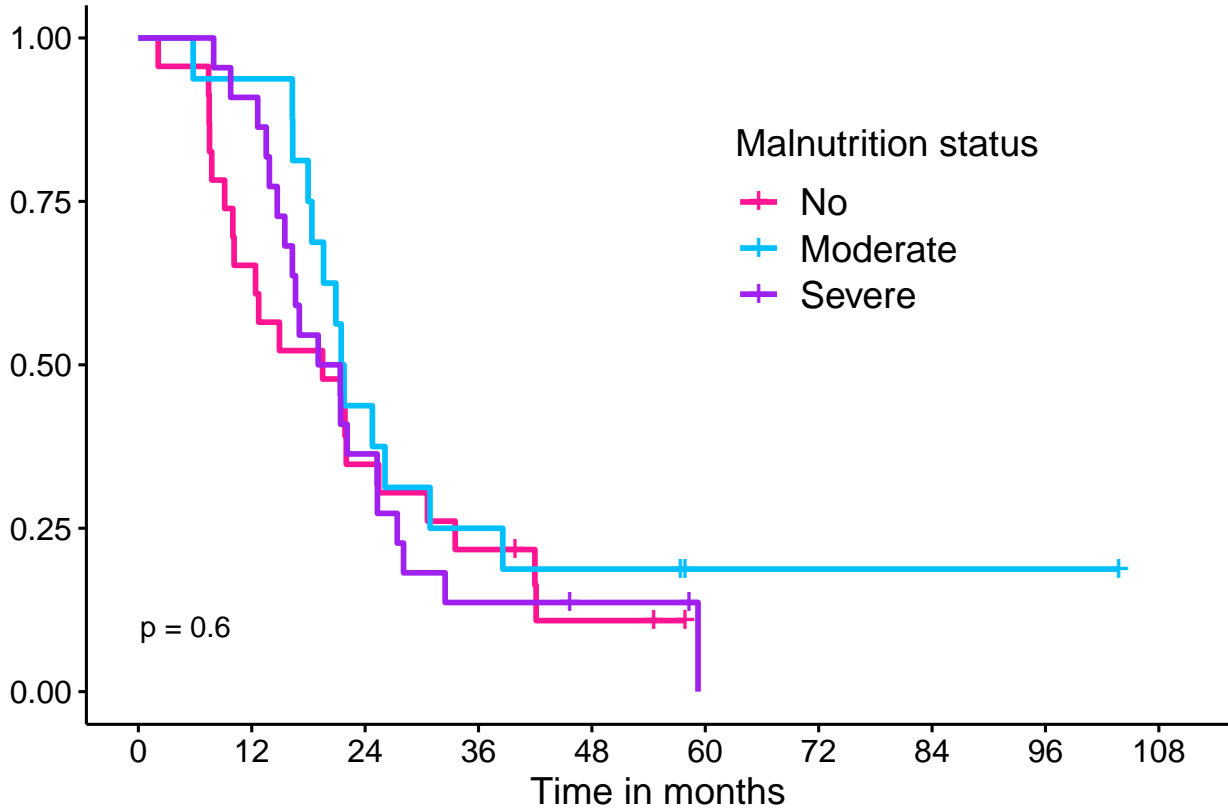

Number at risk

|          |    |    |   |   |   |   |   |   |   |   |
|----------|----|----|---|---|---|---|---|---|---|---|
| No       | 23 | 15 | 8 | 5 | 2 | 0 | 0 | 0 | 0 | 0 |
| Moderate | 16 | 15 | 7 | 4 | 3 | 1 | 1 | 1 | 1 | 0 |
| Severe   | 22 | 20 | 8 | 3 | 2 | 0 | 0 | 0 | 0 | 0 |

Supplement: Supplementary file 14 — Additional file 14. Nutritional status and overall survival of cancer patients. Kaplan-Meier curve illustrating overall survival in cancer patients with no, moderate, and severe malnutrition. Patients that received neoadjuvant chemotherapy (N = 6), one patient with metastasis at the time of diagnosis and one patient with non-standard treatment regime were excluded. NA = 3. [file 12885_2024_12320_MOESM14_ESM.pdf]

Survival probability

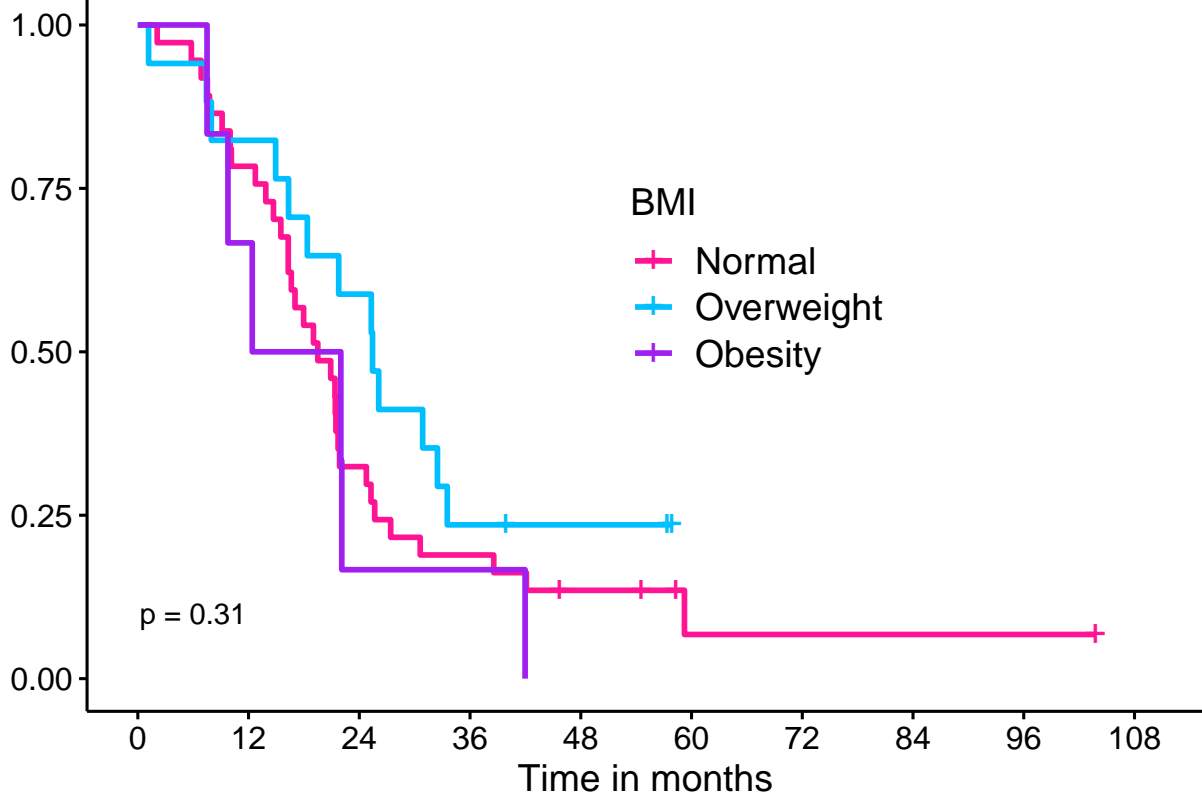

Number at risk

|            |    |    |    |   |   |   |   |   |   |   |
|------------|----|----|----|---|---|---|---|---|---|---|
| Normal     | 37 | 29 | 12 | 7 | 4 | 1 | 1 | 1 | 1 | 0 |
| Overweight | 17 | 14 | 10 | 4 | 3 | 0 | 0 | 0 | 0 | 0 |
| Obesity    | 6  | 4  | 1  | 1 | 0 | 0 | 0 | 0 | 0 | 0 |

Supplement: Supplementary file 15 — Additional file 15. BMI and overall survival of cancer patients. Kaplan-Meier curve illustrating overall survival in cancer patients with normal weight (BMI 18.5–25), overweight (BMI 25–30, and obesity (BMI > 30) according to WHO’s classification. Patients that received neoadjuvant chemotherapy (N = 6), one patient with metastasis at the time of diagnosis and one patient with non-standard treatment regime were excluded. Underweight patients (N = 3, BMI < 18.5) are not illustrated. NA = 1. [file 12885_2024_12320_MOESM15_ESM.pdf]

## Phenylalanine and overall survival

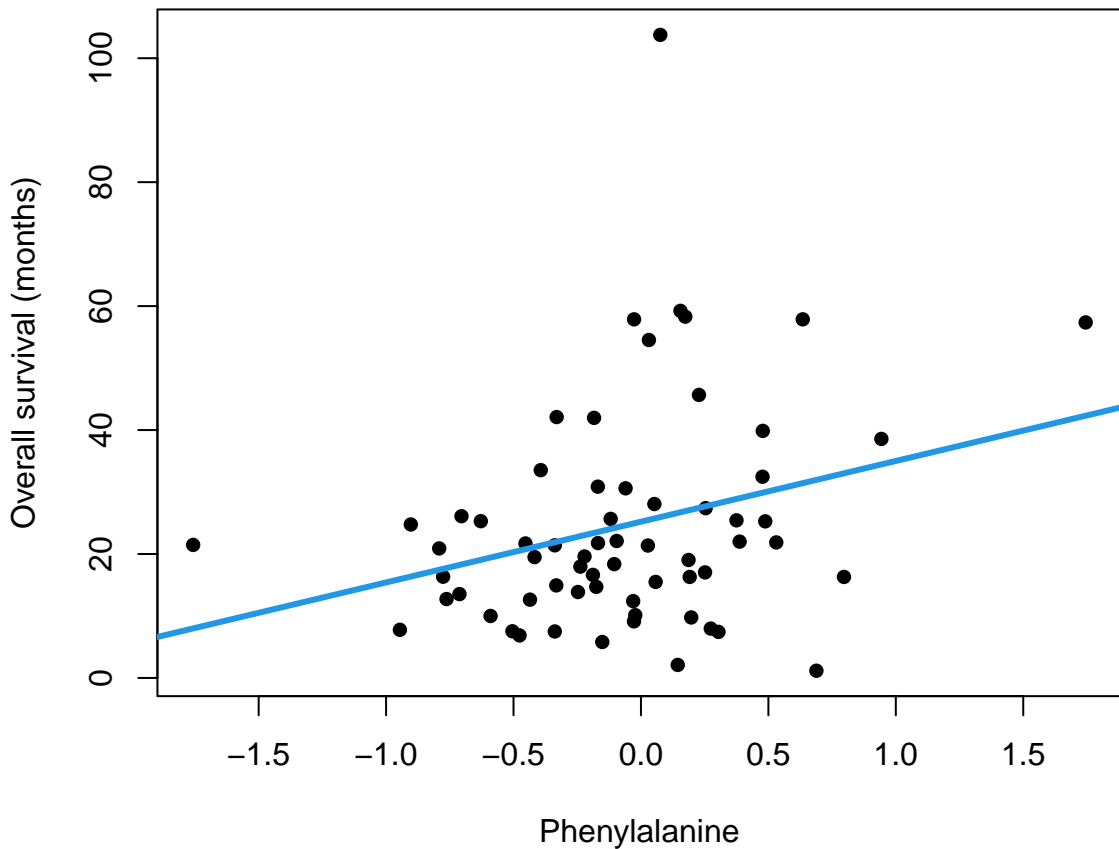

Supplement: Supplementary file 16 — Additional file 16. Plasma Phenylalanine and overall survival of cancer patients. Scatter plot illustrating the association between levels of plasma Phenylalanine and overall survival in cancer patients. Each dot represents a sample. The line represents the direction of the correlation. Patients that received neoadjuvant chemotherapy (N = 6), one patient with metastasis at the time of diagnosis and one patient with non-standard treatment regime were excluded. [file 12885_2024_12320_MOESM16_ESM.pdf]
